# Hepatitis B Virus Infection and Its Determinants among Pregnant Women in Ethiopia: A Systematic Review and Meta-Analysis

**DOI:** 10.1155/2020/9418475

**Published:** 2020-06-11

**Authors:** Addisu Alehegn Alemu, Liknaw Bewket Zeleke, Bewket Yesarah Aynalem, Getachew Mullu Kassa

**Affiliations:** College of Health Sciences, Debre Markos University, Debre Markos, Ethiopia

## Abstract

**Background:**

Hepatitis B virus (HBV) is an infectious and a global public health problem. The prevalence of HBV infection among pregnant women is between 2.3% and 7.9%. HBV infection during pregnancy is associated with prenatal transmission to the fetus. HBV has an effective vaccine which reduces up to 96% of the transmission. Although different studies were conducted in Ethiopia, none of them showed the national prevalence of HBV infection among pregnant women. Therefore, this study was conducted to determine the pooled prevalence of HBV and its associated factors in Ethiopia.

**Methods:**

We followed the Preferred Reporting Items for Systematic Reviews and Meta-Analyses (PRISMA) guidelines for articles. All observational published studies were retrieved using relevant search terms in Google Scholar, African Online Journal, CINAHL, and PubMed databases. Newcastle-Ottawa assessment checklist for observational studies was used for critical appraisal of the included articles. The meta-analysis was done with STATA version 14 software. The *I*^2^ statistics were used to test heterogeneity whereas Begg's and Egger's tests were used to assess publication bias. Odds ratio (OR) with a 95% confidence interval (CI) was presented using the forest plot.

**Results:**

A total of twenty-three studies were included in this systematic review and meta-analysis. The pooled prevalence of HBV in Ethiopia was 4.75% (95% CI: 4.06, 5.44). The subgroup analysis showed a higher prevalence of HBV infection among pregnant women in Gambella (7.9%) and the lowest in Southern Nations, Nationalities, and Peoples' Region (SNNPR) (2.3%). Associated factors with HBV infection include history of multiple sexual partner (OR = 6.02 (95%CI = 3.86, 9.36)), blood transfusion history (OR = 5.71 (95%CI = 3.25, 10.04)), abortion history (OR = 3.58 (95%CI = 2.10, 6.09)), and history of body tattoo (OR = 2.83 (95%CI = 1.55, 5.17)).

**Conclusions:**

HBV infection among pregnant women is a common public health problem in Ethiopia. Multiple sexual partners, abortion history, blood transfusion history, and body tattoo were significantly associated with HBV infection. Policies and strategies should focus on factors identified in this study to improve the prevention of HBV among pregnant women.

## 1. Background

Hepatitis B virus (HBV) infection (acute and chronic) is one of the most common causes of human liver disease, and most people usually remain unaware of their infection status and present when the disease is advanced [1, 2]. HBV is an important global public health problem [2, 3], in which 350 million people had been diagnosed for chronic HBV infection and 686,000 people die each year from its complications, which include cirrhosis and hepatocellular carcinoma [2]. It is among the tenth killer disease worldwide [4–6] and named as “silent killer” [7]. It is mainly affecting developing countries [4, 8–11]. Globally, HBV infection endemicity is classified as high (≥8%), intermediate (2-7%), and low (<2%) depending on its prevalence [12]. The HBV classification in Ethiopia is intermediate [13] and ranges between 2.3% [14] and 14% [12].

People are usually infected with HBV through direct contact with infected blood and other body secretions. However, it can be transmitted from infected mothers to their newborns during pregnancy and delivery [15–17]. The latter is the commonest cause of the HBV infection [5, 18] associated with insufficient knowledge about HBV among pregnant women and the absence of universal screening of all women attending antenatal care [5, 19, 20]. Many women infected with HBV during their pregnancy are at increased risk of transmitting hepatitis B infection to their unborn babies [6, 11, 21], with a 70-90% chance of perinatal acquisition and over 85-90% chance of becoming chronic carriers of the disease, the main reservoir for continued transmission of HBV [6, 22]. In addition to the current practice, universal child vaccination and routine and continuous antenatal screening program reduce its endemicity [11, 13, 23].

There are many studies conducted on the prevalence of HBV and its associated factors in Ethiopia. However, these studies presented inconsistent and inconclusive findings in the prevalence of HBV infection and its associated factors among pregnant women. Therefore, this systematic review and meta-analysis was conducted to assess the prevalence and associated factors of HBV infection in Ethiopia using available published evidence. The findings of this study will provide input in the design of proper strategies to reduce HBV infection among pregnant women.

## 2. Methods

### 2.1. Study Selection

We conducted this systematic and meta-analysis of all observational published studies to assess the pooled prevalence and determinants of HBV infection among pregnant women in Ethiopia. Retrieving of the included studies was done using different databases such as Google Scholar, African Online Journal, CINAHL, and PubMed without restricting study period. We carried out the selection of previous studies to include in the current study following the Preferred Reporting Items for Systematic Reviews and Meta-Analysis (PRISMA) guideline [24].

### 2.2. Inclusion Criteria of the Studies

*Study design*. All observational published studies in the English language and reported prevalence/magnitude and associated factors of HBV infection among pregnant women in Ethiopia were included.

*Study period*. All studies conducted from January 2004 to May 2018 and published from 2005 to January 1, 2020, were included in this review.

*Participants*. Women who had become pregnant at least once before or during each study.

*Exposure*. The magnitude/prevalence and determinants of HBV infection. Determinants are general characteristics that might increase or decrease the chance of women infected with HBV. It includes history of abortion, blood transfusion, multiple sexual partners, and body tattoo.

*Outcome*. Mothers infected with HBV.

### 2.3. Search Strategy

Prevalence; seroprevalence; seroepidemiology; magnitude; hepatitis B Virus; Viral hepatitis; HBV; Hepatitis B surface antigen; HBsAg; Viral liver disease; pregnant woman and Ethiopia were MeSH terms used for searching using “OR” and “AND” Boolean operators. Additionally, we have checked the identified studies' references on the databases to find the potentially eligible studies but missed during the initial searching.

### 2.4. Data Extraction

Data for this study were extracted from the previous studies using an extraction form developed on the Excel sheet by the authors. Two of the authors (AAA and LBZ) participated in extracting data from the included studies. For all included studies, we have considered and recorded the publication year, author name, study design, sample size, and exposure characteristics, i.e., prevalence, history of abortion, blood transfusion, multiple sexual partners, and body tattoo. Data extraction was done using a structured form on Microsoft Excel.

### 2.5. Risk of Bias (Quality Assessment)

An intensive assessment of all the articles included in this study was done by the two authors (AAA and LBZ). Newcastle-Ottawa assessment checklist [25] for observational studies was applied for assessing the quality of each study considered in this research. The tool has three sections; the first section (methodological assessment) and the second section (comparability evaluation) are rated up to five and three stars, respectively, whereas the third section was used for assessing statistical analysis and outcome for each included study. All the included articles assessed through the tool and studies that scored ≥6 were taken with high quality. There was a joint discussion between the reviewers for uncertainty.

### 2.6. Data Synthesis and Statistical Analysis

The data entry was done through Microsoft Excel and exported to STATA version 14 software for analysis. Figures and tables were used to show the summarized and descriptive results. We have also conducted a meta-analysis to assess the pooled prevalence and determinants of the outcome variable (HBV infection). We have estimated adjusted odds ratios with their confidence intervals to measure the association. The random-effect model was considered to determine the pooled prevalence and determinants of HBV infection among pregnant women in Ethiopia due to the heterogeneity by study design and study regions/areas. *I*^2^ statistics of 25, 50, and 75% were used to declare low, moderate, and high heterogeneity, respectively [26]. We had subgroup analysis by region because of the heterogeneity among the included studies to estimate the pooled prevalence. We have also checked publication bias using Egger's and Begg's tests, and a *p* value of less than 0.05 was used to declare its statistical significance [27, 28].

## 3. Results

### 3.1. Study Selection

**Figure 1:**
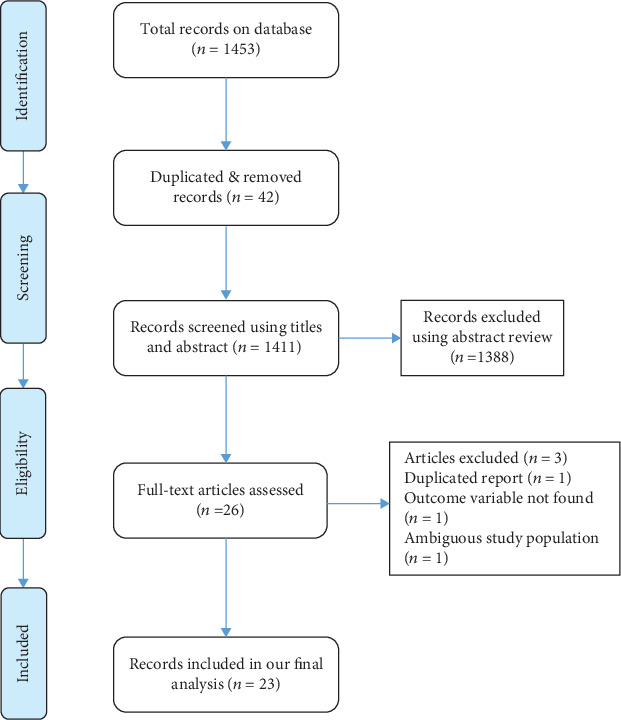
PRISMA diagram identifying studies utilized for systematic and meta-analysis of HBV infection among pregnant women in Ethiopia.

All published observational studies on HBV infection among pregnant women in Ethiopia were included in this systematic and meta-analysis study. A total of 1453 articles were found on the databases, 42 of which were duplicated and removed through title screening. After screening of all the retrieved records, 1388 articles were excluded. A total of 26 full-text studies were assessed for eligibility; finally, 23 studies were included in the meta-analysis of this study (Figure 1).

### 3.2. Characteristics of Included Studies

Twenty-three of the studies included in the final analysis were cross-sectional [11, 14, 23, 24, 29–47]. The studies used health facility-based HBV infection data among pregnant women which were collected from 2002 to 2018 in the respective health institutions. Seven articles were conducted at the Southern Nations, Nationalities, and Peoples' Region (SNNPR) [11, 14, 29, 31, 35, 39, 47], five in Amhara region [23, 24, 30, 32, 36], five in Oromia region [33, 37, 38, 41, 44], and three in Addis Ababa [34, 41, 42], whereas one in Tigray [46], Harar [43], and Gambella [45]. The sample size of included studies ranges from a minimum of 165 pregnant women in SNNPR [39] to 580 in Oromia [38]. Overall, a total of 7,860 pregnant women were included in this review. The quality score of the included articles ranges from 6 to 9 (Table 1).

### 3.3. Prevalence of HBV among Pregnant Women in Ethiopia

**Figure 2:**
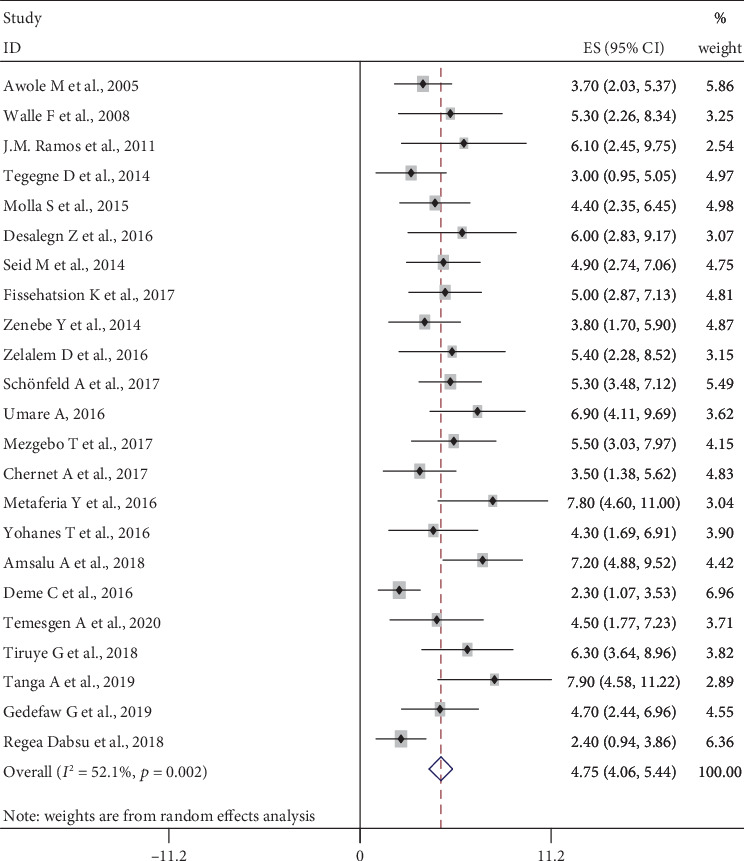
The pooled prevalence of HBV infection among pregnant women in Ethiopia.

The prevalence of HBV infection among the included studies ranges from a minimum of 2.3% (95% CI: 1.07, 3.53) in southern Ethiopia [14] to a maximum of 7.9% (95% CI: 4.58, 11.22) in Gambella Hospital, Southwestern, Ethiopia [45]. The pooled prevalence of HBV infection among pregnant women in Ethiopia was 4.75% (95% CI: 4.06, 5.44) based on the random-effect model analysis. Heterogeneity test showed presence of medium heterogeneity, *I*^2^ = 52.1% and *p* value = 0.002. There was also significant publication bias detected, *p* value ≤ 0.001 (Figure 2).

### 3.4. Subgroup Analysis

Subgroup analysis was conducted by different study characteristics. The subgroup analysis by region showed the highest prevalence of HBV infection in Gambella regional state, 7.9% (95% CI: 4.58, 11.22), and the lowest in SNNPR, 2.3% (95% CI: 1.07, 3.53), even if one article was included from each region. The pooled prevalence of HBV infection in Amhara, Oromia, Addis Ababa, Tigray, Harar, and Gambella regions was 4.53% (95% CI: 3.52, 5.54), 4.47% (95% CI: 2.92, 6.02), 4.42% (95% CI: 2.73, 6.12), 5.50% (95% CI: 3.03, 7.97), 6.30% (95% CI: 3.64, 8.96), and 7.90 (4.58, 11.22), respectively. The prevalence of HBV infection among pregnant women before the year 2015 was 4.70 (95% CI: 4.12, 5.29), and it increased to 4.78% (95% CI: 3.09, 6.47) after 2015. For publication bias confirmed by the Egger test, the Duval and filled analyses were conducted to fill with unpublished studies (Table 2).

### 3.5. Factors Associated with HBV Infection

#### 3.5.1. History of Multiple Sexual Partners

**Figure 3:**
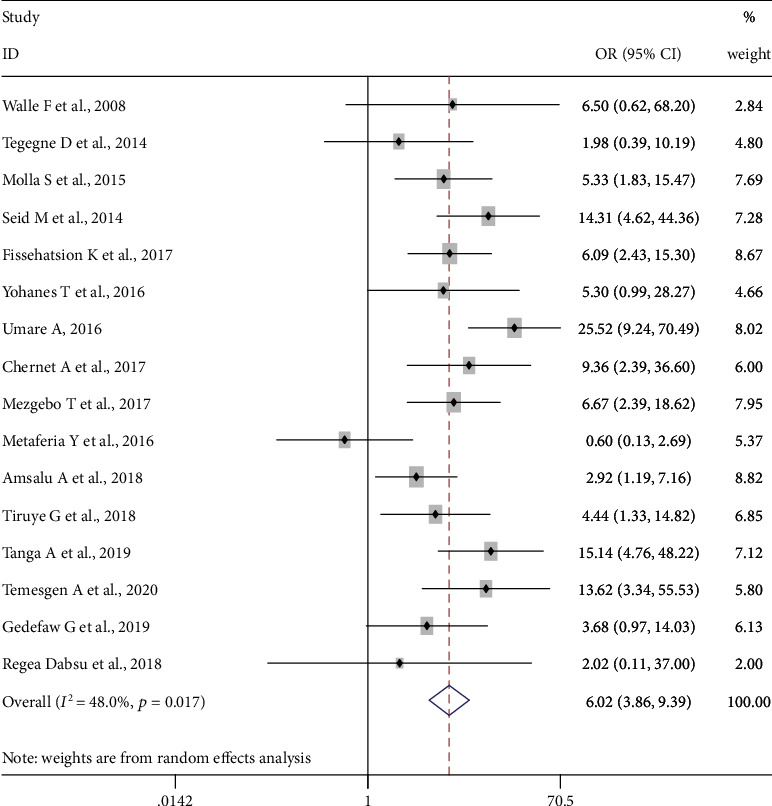
Forest plot on the effect of having multiple sexual partners on HBV infection.

Sixteen studies, 5111 pregnant women, were included in this category of meta-analysis. Ten of the included studies [11, 23, 29, 30, 42–47] showed the presence of association of multiple sexual partners with a higher risk of HBV infection. The meta-analysis showed a strong association between the history of multiple sexual partners and HBV infection, OR = 6.02 (95% CI: 3.86, 9.36). The heterogeneity test showed statistical evidence of heterogeneity, *I*^2^ = 48% and *p* value = 0.017. Begg's and Egger's tests for publication bias also showed no statistical evidence of publication bias, *p* value = 0.368 and *p* value = 0.370, respectively (Figure 3).

#### 3.5.2. History of Blood Transfusion

**Figure 4:**
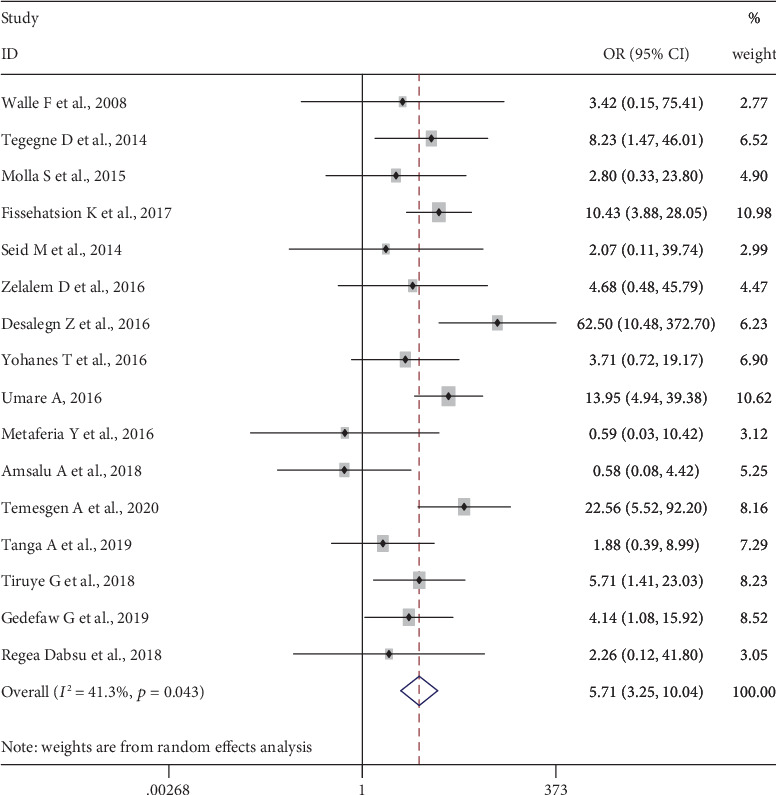
Forest plot on the effect of history of blood transfusion on HBV infection.

Sixteen studies, 4910 pregnant women, were included in this category of meta-analysis [11, 23, 24, 29–35, 41–45]. Seven of the included studies [11, 32, 34, 41–44] showed a significant association between history of blood transfusion and a higher risk of HBV infection. The pooled meta-analysis showed higher odds of HBV infection among pregnant women who had a history of blood transfusion than those who had no history of blood transfusion, OR = 5.71 (95%CI = 3.25, 10.04). Significant heterogeneity (*I*^2^ = 41.3%, *p* value = 0.043) was found, whereas Begg's tests showed no statistical evidence of publication bias, *p* value = 0.150 (Figure 4).

#### 3.5.3. History of Abortion

**Figure 5:**
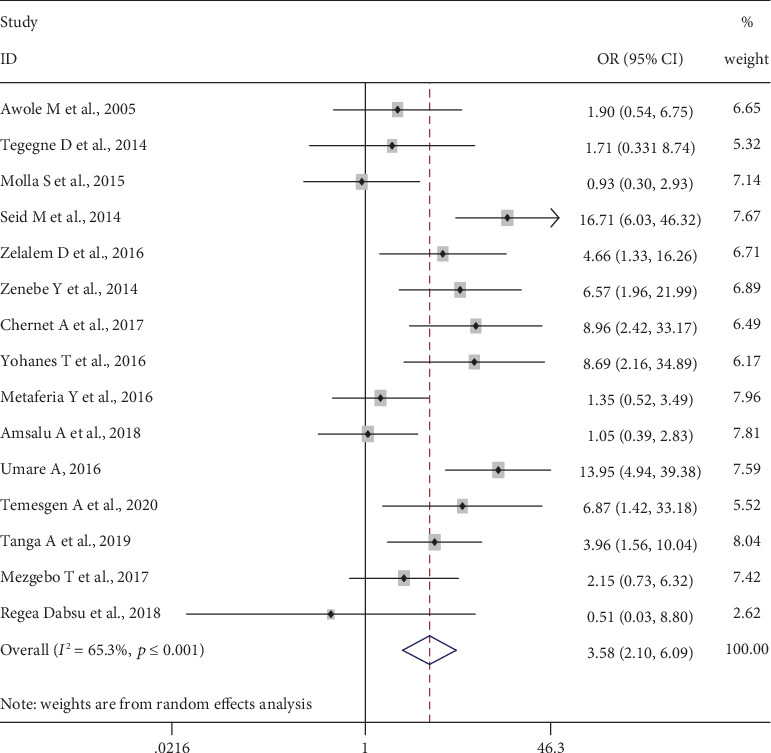
Forest plot on the effect of history of abortion on HBV infection.

Fifteen studies [11, 23, 29–31, 33–37, 41, 44, 45, 47], 4,854 pregnant women, were included to determine the association of history of abortion and HBV infection. The pooled meta-analysis showed that pregnant women who had an abortion are more than three or more times more likely to be infected with HBV, OR = 3.58 (95% CI: 2.10, 6.09). Heterogeneity test showed evidence of high heterogeneity, *I*^2^ = 65.3% and *p* value ≤ 0.001. However, there was nonsignificant publication bias, Begg′s test = 0.961 and Egger′s test = 0.855 (Figure 5).

### 3.6. Body Tattoo

**Figure 6:**
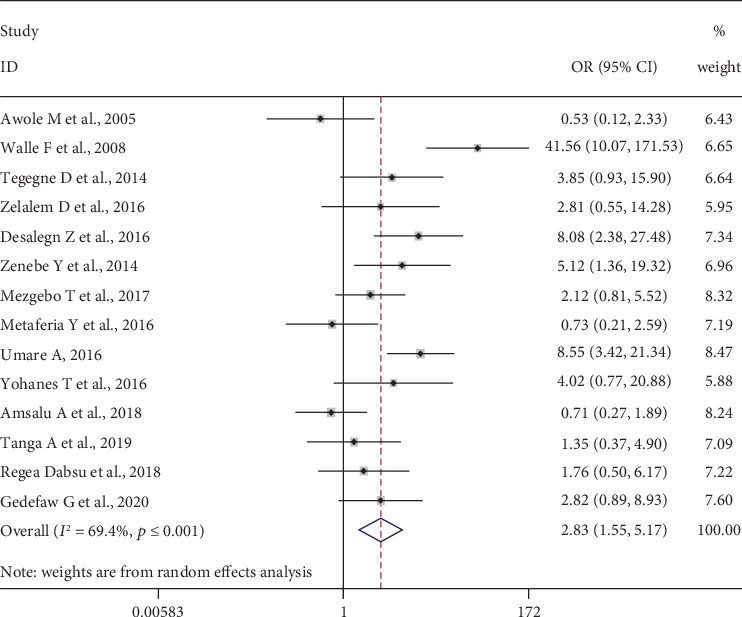
Forest plot on the effect of body tattooing on HBV infection.

Fourteen studies [24, 29, 31–37, 41, 44–46], 4336 pregnant women, were included to determine the association of history of abortion and HBV infection. The pooled meta-analysis showed that pregnant women who had an abortion are more than three or more times more likely to be infected with HBV, OR = 2.83 (95% CI: 1.55, 5.17). Heterogeneity test showed evidence of high heterogeneity, *I*^2^ = 69.4% and *p* value ≤ 0.001. However, there was nonsignificant publication bias, Begg′s test = 0.956 and Egger′s test = 0.838 (Figure 6).

## 4. Discussion

HBV infection screening and treatment among pregnant women in addition to universal newborn vaccination [48, 49] are important to prevent perinatal mortality [38] whereas screening pregnant women for hepatitis B infection based on risk factors may not be effective [50]. Vaccination of neonates of HBV-positive mothers within 24 hours of delivery prevents 90-95% of transmission [2]. Moreover, health education on routes of transmission has paramount importance on HBV infection prevention [29, 51].

We have investigated the prevalence and associated factors of HBV infection among pregnant women in Ethiopia, considering it will be an input for the government, nongovernmental organizations, and stakeholders who want to work on limiting and reducing it. This systematic review and meta-analysis found that the pooled national level prevalence of HBV infection among pregnant women was 4.75% (95% CI: 4.06, 5.44), ranged as intermediate according to the WHO classification [52]. It was almost similar to a meta-analysis study in Ethiopia (4.7%) [13]. This similarity might be due to the fact that both studies are nationwide and have similar study population. However, the prevalence of this study was lower than that of a nationwide study done in other developing countries such as in Ghana (12.3%) [3], Cameroon (11.2%) [53], and Nigeria (13.6%) [54]. It might be attributed to the high prevalence of HBV infection among the general population of the countries [13] which is higher than the prevalence of HBV infection of the general population in Ethiopia 7.4% [22]. Generally, its heavy burden of high cost for prevention, treatment, and management [55] makes HBV infection higher in developing countries than in developed countries. This is supported by the studies that showed less prevalence of HBV infection among pregnant women in developed countries, i.e., Denmark (0.26%) [49], Turkey (1.74%) [56], and Spain (0.1%) [57]. This lower prevalence might be due to universal screening and vaccination; universal screening for hepatitis B has increased vaccination coverage up to 96% among pregnant women [49].

Furthermore, this systematic review and meta-analysis found that pregnant women who had a history of blood transfusion were more likely to be infected with HBV, which is supported by the studies done in Ethiopia [36], Pakistan [58], Nigeria [59], Congo [58], Cameroon [60], Uganda [61], and Sudan [62]. This might be attributed to the incomplete screening coverage before donation [63].

This study also identified pregnant women who had multiple sexual partners significantly associated with HBV infection in Ethiopia. This is similar to studies done in Ethiopia [44], Nigeria [59], India [64], and South Sudan [65]. It might be due to HBV transmitted through sexual intercourse, and when it is unprotected and is done with multiple partners, the transmission rate will be increased. Similarly, this study showed pregnant women who had abortion history are more likely to be infected with HBV which is also in line with other previous studies done in Ethiopia [11], Kenya [66], Nigeria [67], Sudan [68], and Uganda [61] in which it might be attributed by the fact that most of the common causes of abortion is unplanned pregnancy and with unprotected sexual intercourse that makes them prone to communicable disease.

Moreover, this systematic review and meta-analysis also revealed that pregnant women who had a tattoo on their body were infected with HBV more likely than those who had not, similarly with the studies done in Ethiopia [36, 41], Sudan [68], Turkey [51], Ghana [51], Nigeria [59], and Rwanda [69]. It might be due to a gap in the cleaning and sterilizing of materials for the procedure. The outcome variable may be affected by other variables not included in this study.

## 5. Conclusions

The prevalence of HBV infection among pregnant women in Ethiopia is intermediate though variations are observed across regions. A higher risk of HBV infection was observed among pregnant women who had a history of abortion, who had a blood transfusion, who had a tattoo on their body, and who had multiple sexual partners. This study identified the magnitude and some of the factors associated with HBV infection. Therefore, the Ministry of Health and other concerned organizations should take note of this evidence to prevent further infection of HBV.

## Figures and Tables

**Table 1:** Descriptions of the studies included in the current study.

Study ID	Study year	Prevalence	Sample	Region	Quality
Zenebe Y, 2014 [36]	2014	3.8	318	Amhara	9
Desalegn Z, 2016 [41]	2014	6	215	Addis Ababa	8
Gedefaw G, 2019 [32]	2018	4.7	338	Amhara	8
Chernet A, 2017 [47]	2015	3.5	289	SNNPR	9
Tiruye G, 2018 [43]	2017	6.3	320	Harar	7
Tanga A, 2019 [45]	2017	7.9	253	Gambella	9
Tegegne D, 2014 [34]	2012	3	265	Addis Ababa	6
Ramos JM, 2011 [39]	2011	6.1	165	SNNPR	9
Molla S, 2015 [30]	2013-2014	4.4	384	Amhara	9
Umare A, 2016 [44]	2015	6.9	318	Oromia	9
Dabsu R, 2018 [33]	2018	2.4	421	Oromia	7
Temesgen A, 2020 [11]	2017	4.5	222	SNNPR	8
Amsalu A, 2018 [29]	2015-2016	7.2	475	SNNPR	6
Awole M, 2005 [37]	2002-2003	3.7	493	Oromia	7
Walle F, 2008 [24]	2004	5.3	209	Amhara	7
Desalegn Z, 2016 [40]	2014	5.4	202	Oromia	9
Yohanes T, 2016 [31]	2015	4.3	232	SNNPR	8
Fissehatsion K, 2017 [42]	2014	5	403	Addis Ababa	6
Metaferia Y, 2016 [35]	2015	7.8	269	SNNPR	8
Seid M, 2014 [23]	2014	4.9	385	Amhara	7
Mezgebo T, 2017 [46]	2015	5.5	328	Tigray	6
Schönfeld A, 2017 [38]	2014-2015	5.3	580	Oromia	7
Deme C, 2016 [14]	2016	2.3	574	SNNPR	7

**Table 2:** Subgroup analysis of HBV infection among pregnant women by study year and region.

Study year	Number of studies	Prevalence (95% CI)	*p* value	*I* ^2^ (%)
In 2015 & before	16	4.70 (4.12, 5.29)	0.481	0.0
After 2015	7	4.78 (3.09, 6.47)	≤0.001	78.2
Region				
SNNPP	7	4.87 (3.17, 6.57)	≤0.001	72.4
Oromia	5	4.47 (2.92, 6.02)	0.021	65.5
Amhara	5	4.53 (3.52, 5.54)	0.930	0.0
Addis Ababa	3	4.42 (2.73, 6.12)	0.217	34.5
Tigray	1	5.50 (3.03, 7.97)		
Gambella	1	7.90 (4.58, 11.22)		
Harar	1	6.30 (3.64, 8.96)		

## Data Availability

Data used in the current study are available from authors of each study upon request.

## Abbreviations

HBV: Hepatitis B virus
CI: Confidence interval
OR: Odds ratio
SNNPR: Southern Nations, Nationalities, and Peoples' Region
WHO: World Health Organization
PRISMA: The Preferred Reporting Items for Systematic Reviews and Meta-Analysis
SE: Standard error.

## Supplementary Materials

Supplementary Materials PRISMA NMA checklist of items to include when reporting a systematic review involving a network meta-analysis.
